# Performance of a Handheld Ultrasound Device to Assess Articular and Periarticular Pathologies in Patients with Inflammatory Arthritis

**DOI:** 10.3390/diagnostics11071139

**Published:** 2021-06-22

**Authors:** Giulia Corte, Sara Bayat, Koray Tascilar, Larissa Valor-Mendez, Louis Schuster, Johannes Knitza, Filippo Fagni, Georg Schett, Arnd Kleyer, David Simon

**Affiliations:** 1 Department of Internal Medicine 3, Friedrich-Alexander University (FAU) Erlangen-Nuremberg and Universitätsklinikum Erlangen, Ulmenweg 18, 91054 Erlangen, Germany; giulia.corte@uk-erlangen.de (G.C.); sara.bayat@uk-erlangen.de (S.B.); koray.tascilar@uk-erlangen.de (K.T.); larissa.valormendez@uk-erlangen.de (L.V.-M.); louis.schuster@uk-erlangen.de (L.S.); johannes.knitza@uk-erlangen.de (J.K.); filippo.fagni@extern.uk-erlangen.de (F.F.); georg.schett@uk-erlangen.de (G.S.); arnd.kleyer@uk-erlangen.de (A.K.); 2 Deutsches Zentrum fuer Immuntherapie (DZI), FAU Erlangen-Nuremberg and Universitätsklinikum Erlangen, Ulmenweg 18, 91054 Erlangen, Germany

**Keywords:** medical ultrasound, handheld ultrasound device, musculoskeletal ultrasound, inflammatory arthritis

## Abstract

The purpose of this study was to assess the accuracy and performance of a new handheld ultrasound (HHUS) machine in comparison to a conventional cart-based sonographic machine in patients with inflammatory arthritis (IA). IA patients with at least one tender and swollen joint count were enrolled. US was performed on the clinically affected joints using a cart-based sonographic device (Samsung HS40) and a HHUS device (Butterfly iQ). One blinded reader scored all images for the presence of erosions, bony enlargement, synovial hypertrophy, joint effusion, bursitis, tenosynovitis, and enthesitis. Synovitis was graded (B mode and power Doppler (PD)) by the 4-level EULAR-OMERACT scale. To avoid bias by the blinded reader, we included 67 joints of two healthy volunteers in the evaluation. We calculated the overall concordance and the concordance by type of joint and pathological finding. We also measured the time required for the US examination per joint with both devices. Thirty-two patients (20 with RA, 10 with PsA, and one each with gout and SLE-associated arthritis) were included, and 186 joints were examined. The overall raw concordance in B mode was 97% (κappa 0.90, 95% CI (0.89, 0.94)). In B mode, no significant differences were found in relation to type of joint or pathological finding examined. The PD mode of the HHUS device did not detect any PD signal, whereas the cart-based device detected a PD signal in 61 joints (33%). The portable device did not offer any time savings compared to the cart-based device (47.0 versus 46.3 s). The HHUS device was accurate in the assessment of structural damage and inflammation in patients with IA, but only in the B mode. Significant improvements are still needed for HHUS to reliably demonstrate blood flow detection in PD mode.

## 1. Introduction

Medical ultrasound (US) technology is in continuous evolution, not only in terms of image resolution, but also with regard to processing speed, data storage, software features, and, of course, compactness of ultrasound devices. In recent years this development has led to the market entry of handheld ultrasound (HHUS) devices, which are increasingly used in clinical practice thanks to their greater portability and significantly lower purchase cost compared to conventional cart-based US devices [1]. Because HHUS devices are affordable and simple to handle, a growing number of physicians will have convenient access to diagnostic ultrasound in clinical practice, even in rural areas or developing countries [1,2].

HHUS devices have so far been established mainly in internal medicine and emergency medicine, proving to be particularly suitable for emergency pre-hospital examinations [3], bedside examinations [4], and US-guided punctures [5]. Some studies have already shown the good applicability of HHUS devices for musculoskeletal ultrasound (MSUS) examinations, e.g., for hemarthrosis and local degenerative changes such as tendinopathies. [6,7,8,9]. However, the use of HHUS devices in rheumatology to systematically assess structural joint damage and inflammatory extent in inflammatory arthritis (IA) has not been evaluated to date.

MSUS has also become an established imaging modality in clinical practice in rheumatology [10]. There are several reasons for this, such as non-invasiveness, high sensitivity to detect pathologies at low cost [11,12], and simple application also in ultrasound-guided joint punctures [13]. Despite these advantages, MSUS is still not used by all rheumatologists in clinical practice, as often only a limited number of devices are accessible due to the high acquisition costs and necessary accommodation space [14,15,16]. These limitations could be at least partly overcome by the use of HHUS devices, permitting a better and faster assessment in patients with IA.

To address the usefulness of HHUS in the assessment of inflammatory arthritis (IA), we conducted a prospective study to assess the accuracy and performance of a new HHUS machine in comparison to a conventional cart-based sonographic machine in patients with IA. We investigated whether HHUS devices are suitable to assess articular and periarticular inflammation as well as structural damage in patients with IA. Furthermore, we evaluated whether the use of a HHUS device saves time in the assessment of the joints.

## 2. Materials and Methods

### 2.1. Recruitment of Participants

**Figure 1:**
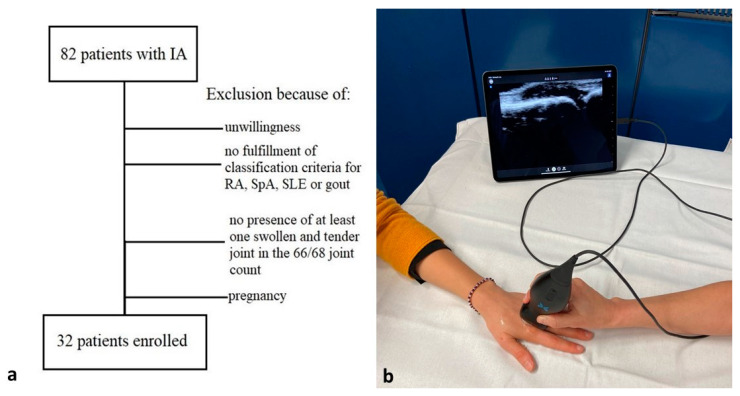
Study flowchart and used handheld US equipment. Depiction of recruitment flowchart (**a**) and the handheld portable US device Butterfly iQ (**b**), which was directly connected to a tablet.

We prospectively recruited patients with IA who visited the rheumatology outpatient clinic of the Department of Internal Medicine 3 (Erlangen, Germany) with at least 1 swollen and tender joint in April 2020 (Figure 1). To be included, patients had to also fulfil classification of the respective diseases such as the ACR/EULAR 2010 RA classification criteria [17], the CASPAR classification criteria [18], the EULAR/ACR 2019 SLE criteria [19], or the 2015 Gout Classification Criteria [20]. In all participants, a tender and swollen joint count was performed (66/68 joint count) by 1 experienced rheumatologist (G.C.). In addition, demographic (age, sex), clinical (disease activity scores; treatment), and laboratory data (C-reactive protein, erythrocyte sedimentation rate) were collected. To avoid bias by the blinded reader, who otherwise would have been tempted to identify pathological findings for each examined joint, we also included 67 joints from 2 healthy volunteers in the evaluation. Healthy volunteers were recruited from healthcare staff and were required not to have any history of rheumatic or musculoskeletal disease. Ethical approval (334_18 B) was obtained (9 Oct 2018), and all participants signed informed consent.

### 2.2. Ultrasound Examination

After the clinical examination, the same rheumatologist performed the MSUS examination of the clinically affected joints and corresponding tendons/entheses with both a standard ultrasound machine and a HHUS device, according to the 2017 EULAR standardized procedures for ultrasound imaging in rheumatology [21].

We used one of the most recent HHUS machines, the Butterfly iQ (Butterfly Network, Guilford, CT, USA) (Figure 1). This device is characterized by an innovative transducer technology based on 1 silicon chip (2D array, 9000 micro-machined sensors) instead of piezoelectric crystal technology. This technology enables a significantly lower price, at around 2000 Euros. With only 1 transducer, it is possible to perform US examinations with a wide scan depth array, emulating curved and linear probes (frequency range: 1–10 MHz). The device consists only of a portable and lightweight transducer (0.313 kg), which can be connected directly to any available smartphone or tablet with iOS operating software. In our study, the HHUS device was connected to an iPad Pro (Apple, Cupertino, CA, USA). The mid-range conventional cart-based sonographic machine “Samsung HS40“(Samsung Electronics, Suwon-Shi, Korea) was used for comparison.

The examiner always used the cart-based sonographic device first and the HHUS device immediately thereafter, making sure that exactly the same anatomical structures and pathological findings were represented with both devices. For the MSUS examination with the “Samsung HS40”, a linear probe with a frequency range of 3–16 MHz was used. Different MSK pre-sets for each joint area were already set up by the manufacturer and selected accordingly by the examiner. Adjustments to the US settings (gain, frequency, focus, or pulse repetition frequency (PRF) for the PD mode) were possible and were made, if needed, in order to improve the quality of the images.

For the Butterfly iQ device, the MSK default setting was selected. The Butterfly iQ software did not allow modification of any ultrasound settings except for gain and time-gain-compensation (TGC). The values of the pre-set imaging parameters are not disclosed by the manufacturer. The examination was conducted in B mode and PD mode with both devices.

During the examination of the healthy controls, the examiner also recorded with a stopwatch the time in seconds taken to perform the MSUS examination of each examined joint with both devices. For this evaluation, exactly the same joints were examined and the same standard scans were performed with both devices. Both devices were already present in the examination room and already switched on when the timing was started.

### 2.3. Image Evaluation

All images collected with the HHUS and the cart-based US device were subsequently anonymized, stored in 2 different folders, and separately scored by a blinded reader (S.B.). The reader had several years of experience in MSUS and had completed the MSUS training courses of the German Society of Ultrasound in Medicine (Deutsche Gesellschaft für Ultraschall in der Medizin, DEGUM).

The blinded reader evaluated all images for the presence of 7 different pathological findings: erosions, bony enlargement, synovial hypertrophy, joint effusion, bursitis, tenosynovitis, and enthesitis. If detected, synovitis was graded both in B mode and PD mode in 3 different grades according to the consensus-based scoring system suggested by the EULAR-OMERACT taskforce [22].

### 2.4. Statistical Data Analysis

Demographic and clinical characteristics were analyzed as means and standard deviations for continuous variables and count data/percentages for categorical variables. We calculated the overall concordance between the 2 devices (percentage of observation pairs in which the same rating was given for both devices regardless of anatomical site and pathological finding examined) as well as the agreement by type of joint and type of finding. The Cohen’s kappa coefficient (κappa) was used to measure interrater reliability in the image scoring. This analysis was performed separately for B mode and PD mode.

For data analyses, we used the open-source R software v. 3.5.3 (R Foundation for Statistical Computing, Vienna, Austria; https://www.r-project.org/, accessed 11 November 2020) with the car and emmeans packages.

## 3. Results

### 3.1. Demographics

Thirty-two IA patients (20 females/12 males) and two female healthy participants were included. The mean age of the 32 patients was 58.2 ± 13.7 years. Twenty (62.5%) patients had RA, 10 (31.3%) PsA, one gout (3.1%), and one SLE (3.1%). Further demographic and clinical characteristics of the patients are listed in Table 1.

### 3.2. Ultrasound Examination

Overall, we examined 186 joints and corresponding tendons/entheses. Of that total, 114 were finger or toe joints (61.3%), 32 wrists (17.2%), 20 knees (10.7%), 11 elbows (5.9%), 5 ankles (2.7%), and 4 shoulders (2.2%).

- B mode assessment

**Figure 2:**
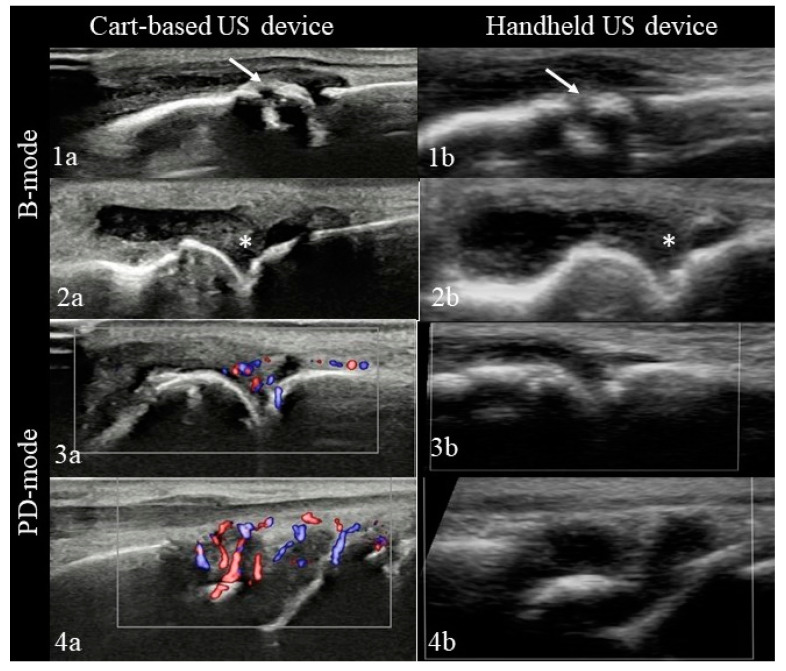
Pathological ultrasound findings depicted by the conventional and the HHUS ultrasound device. Images by the cart-based US machine are presented in the left column, images of the HHUS machine in the right column. B-mode changes of erosions (arrow) (**1a**,**1b**) and synovitis (asterisk) (**2a**,**2b**) in a metacarpophalangeal (MCP) joint displayed by both devices. PD changes of a MCP (**3a**,**3b**) and wrist joint (**4a**,**4b**) could only be visualized by the conventional US device.

In B mode, despite the lower image resolution of the HHUS (Figure 2), we found an overall concordance between the two US devices of 97.1% (Cohen’s Kappa coefficient 0.9 with 95% CI (0.89–0.94)). In B mode, no significant differences were found in relation to the type and size of joint examined or pathological finding evaluated (Table 2).

**Figure 3:**
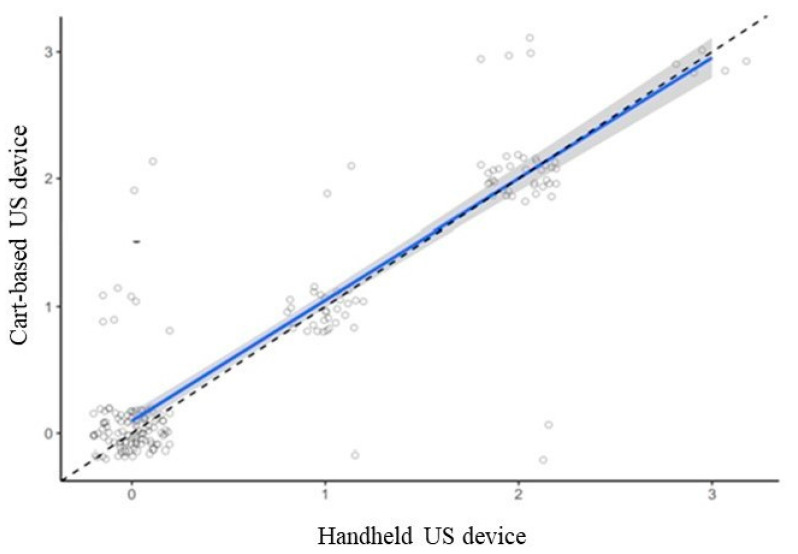
Distribution of OMERACT-EULAR scores for synovitis given by the 2 ultrasound devices. The dashed diagonal line indicates perfect agreement; solid line shows observed agreement. Slightly lower scores were obtained using the HHUS device compared to conventional cart-based device. A small amount of random noise was added to the integer scores to improve visibility.

The concordance in grading synovitis in B mode according to OMERACT EULAR guidelines was 90.3% (Cohen’s kappa coefficient 0.84 with 95% CI (0.76 to 0.91)). As shown in Figure 3, slightly lower grades were given using the portable ultrasound device compared to the cart-based US machine.

- Power-Doppler assessment

In PD mode we found a PD signal in 61 of 186 (33%) joints using the images created by the cart-based device; 31 (51%) were scored as grade 1, 24 (39%) as grade 2, and 6 (10%) as grade 3. The PD mode of the HHUS device did not detect a PD signal in any image, even in the cases, where the conventional US device showed a high-grade PD signal (Figure 2).

- Time comparison between conventional cart-based and handheld US device

The examiner recorded the time needed for the examination of 67 joints of healthy volunteers with both devices. The cart-based device required an average of 46.3 s per joint, while the portable device required a mean time of 47.0 s.

## 4. Discussion

In this study we were able to show for the first time that a HHUS device provides—in B mode—similar results to a mid-range conventional cart-based US device for assessing structural joint damage and inflammation in patients with IA. Despite a maximum frequency of 10 MHz, the HHUS device showed high accuracy in B mode in detecting pathological findings even for small joints, which are often affected in IA patients and for this reason are included in most established US-scoring systems for arthritis [23,24]. Some studies already evaluated the accuracy of HHUS devices for MSUS [6,7,8,9]. However, these studies only focused on the sensitivity assessment of degenerative changes without systematically evaluating pathologies found in IA, e.g., signs of erosions or synovitis. In our work, we used a standardized ultrasound scoring and quantification system that allowed us to make an in-depth comparison of arthritis-specific measurements between a HHUS device and a conventional cart-based US device [22].

However, our study also revealed the absolute inadequacy of the PD mode of the Butterfly iQ device for detecting hypervascularisation in joints and periarticular structures. This limitation seems to be a general problem of HHUS devices at present, thus this shortcoming also applies to other portable devices [9]. Because PD mode has become an essential component of MSUS for assessing and monitoring acute inflammation [25,26,27], improvements in PD mode sensitivity are absolutely necessary to enable the use of HHUS devices in daily rheumatology practice. We believe improvements in the PD technology of HHUS devices in the future are promising, as the use of HHUS devices in rheumatology could help overcome the current limitations of MSUS and facilitate broader access to this technology in rheumatological clinical practice, especially in outpatient settings. Besides its compactness, space-saving benefits, and transport simplicity, the major advantage of HHUS is the significantly lower purchase cost in comparison to conventional cart-based devices. The HHUS device Butterfly iQ used in this study is the most cost-effective portable ultrasound device currently on the market, costing approximately 2000 Euros. The Butterfly iQ, like other HHUS devices, is also equipped with software that allows the exchange of ultrasound images and facilitates remote support of US specialists in the evaluation of sonographic findings. In the future, this approach could even enable rheumatologists to remotely guide and evaluate ultrasound examinations performed by health care specialists or even directly by patients. In this sense, HHUS devices could be used, for example, by general practitioners or dermatologists (e.g., for psoriasis patients) to identify high-risk patients at an early stage and refer them to a rheumatologist. Considering the established role of MSUS in rheumatology as a highly sensitive and non-invasive diagnostic tool [10,11,12,13], the use of HHUS would have a significant positive impact on rheumatological patient care. Furthermore, as already demonstrated for radiology residents [28], the establishment of HHUS devices would also facilitate training in MSUS for rheumatology residents and medical students.

We did not find any time savings when conducting MSUS examinations with a HHUS device itself; this was also a finding reported by another HHUS study [7]. However, for reasons of comparability, in our study, both devices were switched on and directly accessible in the examination room, which is not representative of the typical clinical setting, where a conventional cart-based US device is shared by several specialists and is located in a different room, requiring the movement of patients or transport of the US device. In this case, using the HHUS device would certainly save time thanks to its easy handling and rapid transport.

This study had some limitations. First, the number of patients assessed in this study was relatively small, but as this was a first exploratory study for the use of a HHUS device in IA patients, these numbers were acceptable and in line with other, comparable studies [6,7,8,9]. Second, in our study, image evaluation by the blinded reader was performed with static images, whereas ultrasound video recordings would have more reliably reflected the setting of an ultrasound examination. Lastly, we only tested the Butterfly iQ device, whereas in order to fully establish the applicability of portable ultrasound devices in rheumatology, a study comparing several devices would be necessary. In principle, however, these first data on a HHUS device for use in IA patients are encouraging, considering that portable ultrasound devices are relatively novel on the commercial market and the field is undergoing rapid technological progress [29].

## 5. Conclusions

In this study we were able to show that a handheld ultrasound device is accurate in the assessment of structural joint damage and inflammation in patients with IA, but only in B mode. However, for widespread clinical application in rheumatology, significant improvements will be needed to reliably demonstrate blood flow detection in PD mode.

## Figures and Tables

**Table 1:** Demographic and clinical characteristics.

	Overall	RA	PsA	Other *
N	32	20 (62.5%)	10 (31.3%)	2 (6.2%)
Male/Female	12/20	6/14	5/5	1/1
Age	58.2 ± 13.7	62.1 ± 11.3	55.4 ± 16.2	42.5 ± 3.0
TJ	4.0 ± 3.7	4.1 ± 4.3	3.4 ± 2.2	6.5 ± 5.5
SJ	2.8 ± 2.8	2.9 ± 2.2	2.6 ± 2.0	1.5 ± 0.5
ESR (mm/h)	21.6 ± 17.2	19.2 ± 15.7	24.0 ± 16.9	34.5 ± 24.5
CRP-elevation (%)	11(55.0%)	8 (40.0%)	2 (20.0%)	1 (50.0%)
DAS28-ESR	3.7 ± 1.2	3.6 ± 1.2	3.6 ± 1.2	4.7 ± 0.5
cs-DMARD monotherapy (%)	4 (12.5%)	2 (10.0%)	2 (20.0%)	-
b-/ts-DMARD (%)	17 (53.1%)	12 (60.0%)	4 (40.0%)	1 (50.0%)
cs-DMARD + b-/ts-/DMARD (%)	10 (31.3 %)	6 (30.0%)	3 (30.0%)	1 (50.0%)
Glucocorticoids (%)	6 (18.8%)	4 (20.0%)	1 (10.0%)	1 (50.0%)

* 1 gout patient and 1 SLE patient. RA, rheumatoid arthritis; PsA, psoriatic arthritis; TJ, tender joints in the 66/68 joint count; SJ, swollen joints in the 66/68 joint count; ESR, erythrocyte sedimentation rate; CRP, C-reactive protein; DAS 28-ESR, disease activity score 28-erythrocyte sedimentation rate; cs-DMARD, conventional disease-modifying anti-rheumatic drug; b-/ts- DMARD, biological or targeted synthetic disease-modifying anti-rheumatic drug.

**Table 2:** Concordance between portable US device and conventional US device in B mode.

**Agreement by Site**			
	**N Joints (%)**	**Concordance (%)**	**Kappa 95%CI**
Overall	186	97.1	0.90 (0.89 to 0.94)
Wrist	32 (17.2)	95.5	0.86 (0.77 to 0.93)
Finger/toe joint (MCP, PIP, DIP, MTP)	114 (61.3)	97.4	0.92 (0.88 to 0.95)
Elbows	11 (5.9)	94.8	0.87 (0.75 to 0.97)
Shoulder	4 (2.2)	100.0	1.00 (NA to NA) *
Knee	20 (10.7)	97.9	0.96 (0.90 to 1.00)
Ankle	5 (2.7)	100.0	1.00 (NA to NA) *
**Agreement by pathological finding**			
Joint effusion		95.1	0.81 (0.68 to 0.92)
Synovitis		93.5	0.87 (0.79 to 0.93)
Synovitis OMERACT grade (0– 3)		90.3	0.84 (0.76 to 0.91)
Bone enlargement		98.4	0.88 (0.71 to 1.00)
Erosion		97.8	0.89 (0.77 to 0.89)
Tenosynovitis		97.8	0.83 (0.61 to 0.96)
Entheseopathy		100.0	1.00 (NA to NA) *
Bursitis		100.0	1.00 (NA to NA) *

CI, confidential intervals; MCPs, metacarpophalangeal joints; PIPs, proximal interphalangeal joints; DIPs, distal proximal interphalangeal joints; MTPs, metatarsophalangeal joints; * Estimates unreliable due to low numbers. * Unreliable kappa statistics because of small number of shoulders/ankles examined and small number of enthesopathies and bursitis.

## Data Availability

All relevant data can be found in the manuscript.
